# Ultrasound Assisted Extraction of Polyphenols from Ripe Carob Pods (*Ceratonia siliqua* L.): Combined Designs for Screening and Optimizing the Processing Parameters

**DOI:** 10.3390/foods11030284

**Published:** 2022-01-21

**Authors:** Maria Lisa Clodoveo, Pasquale Crupi, Marilena Muraglia, Filomena Corbo

**Affiliations:** 1 Dipartimento Interdisciplinare di Medicina, Università degli Studi Aldo Moro Bari, 11-70124 Bari, Italy; marialisa.clodoveo@uniba.it; 2 Dipartimento di Farmacia-Scienze del Farmaco, Università degli Studi di Bari, Campus Universitario E. Quagliarello Via Orabona, 4-70125 Bari, Italy; filomena.corbo@uniba.it

**Keywords:** carob kibbles, experimental design, phenolic content, UAE, valorization, HPLC-DAD

## Abstract

Carob pulp has recently received great attention due to its considerable content of polyphenols having a wide range of health promoting effects. In this work, ultrasound assisted extraction was optimized sequentially using a screening Plackett–Burman design and non-standard central composite design coupled to response surface methodology and desirability function statistical tools, to find the best conditions for the extraction of nine polyphenols from carob pods. The gathered mathematical models showed that the highest significant factors influencing the extraction of all compounds were solid–solvent ratio, solvent concentration, and particle size, with the optimal results obtained at values of 0.2 g/mL, 40% ethanol, and 0.3 mm, respectively. Extraction temperature, time, sonication power, and frequency were set at 35 °C, 15 min, 100 W, and 37 kHz, respectively. These parameters help to reduce energy costs and to obtain the best possible extraction of polyphenols.

## 1. Introduction

The species carob (*Ceratonia siliqua* L.) is a slow-growing evergreen tree widely diffused in Mediterranean countries, especially Spain, Morocco, Italy, and Portugal [1]. Carob pods, the fruits of the carob tree, consist of pulp (90%), containing sugars, fibers, amino acids, and minerals, and seeds (10%), principally composed of galactomannans, but also other bioactive compounds [2,3]. The seeds are particularly in demand on the market because they are source of gum (locust beam gum), which is employed as growth medium, thickener, and food stabilizer [4] and they are used to make germ flour proposed as a dietetic human food [5]. Instead, the pulp has limited application, for instance, in chocolate and pastry manufacturing or as leftovers in nutritious animal feed, and thus it has low economic value [6]. 

The pulp has recently received growing attention due to its considerable content of polyphenols, which have been acknowledged to have antioxidant and radical scavenging activity together with potential benefits for human health [7,8,9,10]. In this sense, various methods, including solid–liquid and Soxhlet extraction have been employed for the recovery of polyphenols from carob pods and derived products [11,12]. These conventional techniques often requires large quantity of solvents as well as long processing time and high temperature, which can lead to the degradation of the active compounds [6,8]. 

Over the last decades, up-and-coming alternative extraction techniques have been evolved due to their time-saving and environment-friendly properties with cost-effective output of high quality phenolic extracts [13]. Among them, ultrasound assisted extraction (UAE) is engaging for its simplicity and low cost of equipment [14,15]; it is much quicker than conventional methods, because acoustic cavitations of power US (preferably in the frequencies range 18–100 kHz) cause cell walls disruption, increasing mass transfer and favoring solvent access to the cell content [16]. The efficiency of UAE is generally influenced by several factors, including solvent–solid ratio, solvent type and concentration, particle size, and extraction time and temperature [17]. Many researches have shown that the highest extraction of polyphenols and other bioactive compounds was obtained with ultrasound technology and the application of suitable optimization modelling was essential to identify the optimized extraction conditions [17,18,19]. 

Classical one-factor a time experiments and response surface methodology (RSM) are the optimization techniques typically performed. Conversely to the former time-consuming and laborious approach, consisting in changing only a factor at a time while keeping all the others constant, RSM is a mathematical technique based on the fit of a polynomial equation to the experimental data, which is able to generate statistical models for simultaneously optimizing the single factors together with the possible interactions between the different factors [20]. In the case of several response variables (i.e, different polyphenols), the issue of their concurrent optimization arises. However, “desirability function” (D) is a suitable tool for transforming predicted values for multiple dependent variables into a single overall desirability score [14].

Although various reports about the optimization of polyphenols extraction from carob pods have already been published, the optimal conditions for their recovery are not yet well defined, generating significant differences and not comparable findings among the literature, which needs to be integrated with new results. Therefore, this study aimed at optimizing UAE conditions for the simultaneous extraction of the main phenolic compounds, which were qualitatively and quantitatively characterized by HPLC-DAD analyses, from deseeded carob pods (cv. Amele) using RSM and D tools. To select the most influencing factors, a two-level Plackett–Burman (P.-B.) followed by Central Composite (CCD) experimental designs were performed. 

## 2. Materials and Methods

### 2.1. Plant Materials

The experiment was conducted in 2021 on mature carob pods (*Ceratonia siliqua* L.) of Amele variety, collected from the same tree cultivated in Apulia region (southern Italy). For extraction optimization and polyphenol characterization, the fresh pods were washed, deseeded, cut into small pieces (2–3 cm), and subsequently grinded to a fine powder by an IKA A11 basic homogenizer (IKA, WERKE GMBH & CO.KG, Staufen, Germany).

### 2.2. Chemicals

Formic acid, ethanol, HPLC grade water, and acetonitrile were supplied by Merk Life Science S.r.l. (Milano, Italy). Chlorogenic acid was purchased from Phytolab (Aprilia, Italy). Gallic acid, ferulic acid, 4-coumaric acid, caffeic acid, procyanidin B_1_, procyanidin B_2_, quercitrin, myricitrin, and quercetin were purchased from Extrasynthese (Genay, France) and used as HPLC reference standards.

### 2.3. Ultrasound Assisted Extraction Process

UAE of polyphenols was performed by using an ultrasonic water bath (Elmasonic P 30H, Elma Schmidbauer GmbH, Singen, Germany) operating in continuous mode and equipped with sensors to allow remote control of the power, frequency, time, and temperature. One gram of carob powder, passed through laboratory test sieves of 0.3, 0.5, 1, and 2 mm (Endecotts LTD, London, UK) to obtain uniformly sized particles and carefully weighed (EU-C1200, Gibertini s.r.l., Novate Milanese–Milano, Italy) into 50 mL capped centrifuge tubes, were used in every experiment and the extraction conditions were chosen according to the experimental design. 

After the ultrasound treatment, the extracts were centrifuged at 4000× *g* for 15 min at 5 °C in an EPPENDORF centrifuge 5810R (Hamburg, Germany), filtered through a 0.45 μm syringe cellulose filter, and analyzed by HPLC-DAD.

### 2.4. HPLC-DAD Analysis

HPLC 1260 (Agilent Technologies, Palo Alto, CA, USA), composed of a degasser, quaternary pump solvent delivery, thermostated column compartment, and diode array detector, was employed for the polyphenols analysis. The extracts (3 μL) were injected onto a reversed stationary phase column, Zorbax SB-C18 (Agilent Technologies, Palo Alto, CA, USA) 3.5 μm (150 × 4.6 mm i.d.), protected by a pre-column, Gemini C18 (Phenomenex, Torrance, CA, USA) 5 μm (4 × 2 mm i.d.), and maintained at 40 °C. HPLC separation was carried out through a binary gradient consisting in water/formic acid (99.9:0.1, *v/v*) (solvent A) and acetonitrile (solvent B): 0 min, 10% B; 4 min, 15% B; 8 min, 15% B; 15 min, 30% B; 18 min, 40% B; 22 min, 55% B; 25 min, 55% B; 30 min, 100% B; 32 min, 100% B; 35 min, 10% B. Stop time to 35 min. Finally the column was re-equilibrated with the initial solvent mixture for 5 min. The flow was maintained at 0.8 mL/min. Diode array detection was between 190 and 400 nm, and absorbance were recorded at 360, 330, and 280 nm. 

Positions of absorption maxima (λ_max_), absorption spectra profile, and retention times (RT) were matched with those from pure standards and used for the compounds identification. Quantification of polyphenols was made by using the calibration curves in the concentration range 100–1.25 μg/mL of gallic acid (R^2^ = 0.9975; LOD = 0.094 μg/mL; LOQ = 0.313 μg/mL), caffeic acid (R^2^ = 0.9956; LOD = 0.0094 μg/mL; LOQ = 0.0313 μg/mL), and myricitrin (R^2^ = 0.9974; LOD = 0.094 μg/mL; LOQ = 0.313 μg/mL). The detection limit (LOD) and quantification limit (LOQ) were calculated on the basis of chromatograms and defined as signal-to-noise (six times SD of baseline) ratio of 3 and 10, respectively.

### 2.5. Experimental Design and Statistical Analyses

Two experimental designs were sequentially adopted for both screening and optimizing UAE parameters. At first, a seven-factor and two-level Plackett–Burman (P.-B.) design was performed to investigate the effect of extraction time (X_1_), extraction temperature (X_2_), solid–solvent ratio (X_3_), solvent concentration (X_4_), sonication frequency (X_5_), sonication power (X_6_), and particle size (X_7_) on polyphenols recovery (Table 1). Based on this preliminary screening procedure, critical influencing factors (X_3_, X_4_, and X_7_) were selected and optimized for the extraction of the HPLC-DAD identified polyphenols by using a non-standard Central Composite Design (CCD) coupled to RSM data treatment. Twenty randomized experiments were carried out, with six replicates at the center values to assess the pure error sum of squares and lack of fit test.

Statistical analysis of P.-B. and CCD designs was performed by STATISTICA 12.0 (StatSoft Inc., Tulxa, OK, United States) software package; after testing their normal distribution by Shapiro–Wilk’s W test, the data were transformed using the Box–Cox transformation technique (Figure S1, Supplementary Materials). Regression analyses of the transformed data to fit second-order polynomial equations (quadratic model) were conducted according to the following equation:

Y_i_ = B_0_ + ΣB_i_X_i_ + ΣB_ii_X_i_^2^ + ΣB_ij_X_i_X_j_ (1)

where Y_i_ is the response function of each analyzed polyphenol; B_0_ is a constant coefficient; B_i_ are the regression coefficients of the linear, quadratic, and interactive terms and X_i_, X_j_ represent the independent variables (X_3_, X_4_, and X_7_). 

On the basis of the analysis of variance (ANOVA), the regression coefficients of linear, quadratic, and interaction terms were obtained and the mathematical models was fitted by evaluating the R^2^ and R^2^_adj_ coefficients. Subsequently, a common D was designed in order to predict unique optimum conditions of the extraction process suitable for all the dependent variables. Finally, further experimental extracts, performed under the optimized UAE, were carried out for the model validation.

## 3. Result and Discussion

### 3.1. Qualitative Analysis of Polyphenols in Ripe Carob Pods Extracts

**Figure 1:**
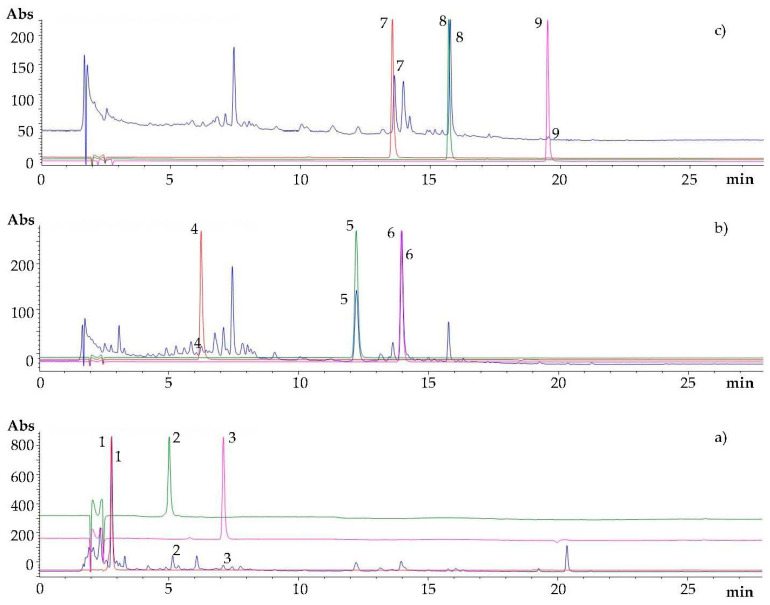
HPLC-DAD chromatograms at (**a**) 280 nm, (**b**) 330 nm, (**c**) 360 nm of ripe carob pulp extract (blue line) compared to reference standards (at 100 μg/mL) of (**1**) gallic acid, (**2**) procyanidin B_1_, (**3**) procyanidin B_2_, (**4**) chlorogenic acid, (**5**) 4-coumaric acid, (**6**) ferulic acid, (**7**) myricitrin, (**8**) quercitrin, (**9**) quercetin.

The main phenolic compounds found in carob pod include phenolic acids, tannins, and flavonoids, whose identity patterns and contents are acknowledged to strongly depend on variety, geographic origin, ripening stage, and extraction methods [6,21]. Figure 1 depicts the HPLC-DAD chromatograms, registered at 280, 330, and 360 nm, of UAE extracts of ripe carob pulp of cv. Amele from Apulia region (southern Italy). The compounds identification was annotated by matching their retention times/elution order and UV absorption spectra to those of available pure standards. As largely reported in the literature [6,22], gallic acid (peak 1, RT = 2.766 min) was the main phenolic compound in the carob pulp (Figure 1a). In addition, chlorogenic acid (peak 4, RT = 6.229 min), 4-coumaric acid (peak 5, RT = 12.205 min), and ferulic acid (peak 6, RT = 13.939 min), exhibiting characteristic UV maxima at 330 nm, were the other phenolic acids identified (Figure 1b).

With regard to the condensed tannins, procyanidin B_1_ (peak 2, RT = 4.995 min) and procyanidin B_2_ (peak 3, RT = 7.091 min), typically present in carob pods [21], were recognized at 280 nm (Figure 1a). Finally, peak 7 at RT = 13.531 min, peak 8 at RT = 15.701 min, and peak 9 at RT = 19.513 min were assigned to flavanols maximally absorbing at 360 nm, namely myricitrin (myricetin-3O-α-rhamnopiranoside), quercitrin (quercetin-3O-α-rhamnopiranoside), and quercetin, respectively (Figure 1c).

### 3.2. Screening of Factors Influencing UAE Efficiency

Generally, the identification of key parameters for the optimization of one or more responses of interest represents a critical step in the development of an experimental design. In particular, a P.-B. saturated design allows the screening of a large number of potential causative factors, yielding unbiased estimates of all main effects in the smallest design possible [23]. 

In this study, a seven-factors (namely, extraction time, extraction temperature, solid–solvent ratio, solvent concentration, sonication frequency, sonication power, and particle size) and two-levels P-B design was developed to assess which operating parameters influence the extraction of polyphenols from ripe carob pulp using UAE. It is worth pointing out that each factor was tested at two most promising levels, chosen on the basis of preliminary experiments and ultrasonic bath specificities, with natural and coded values listed in Table 1. The design matrix of the experimental outcome can be found in Table 2.

**Figure 2:**
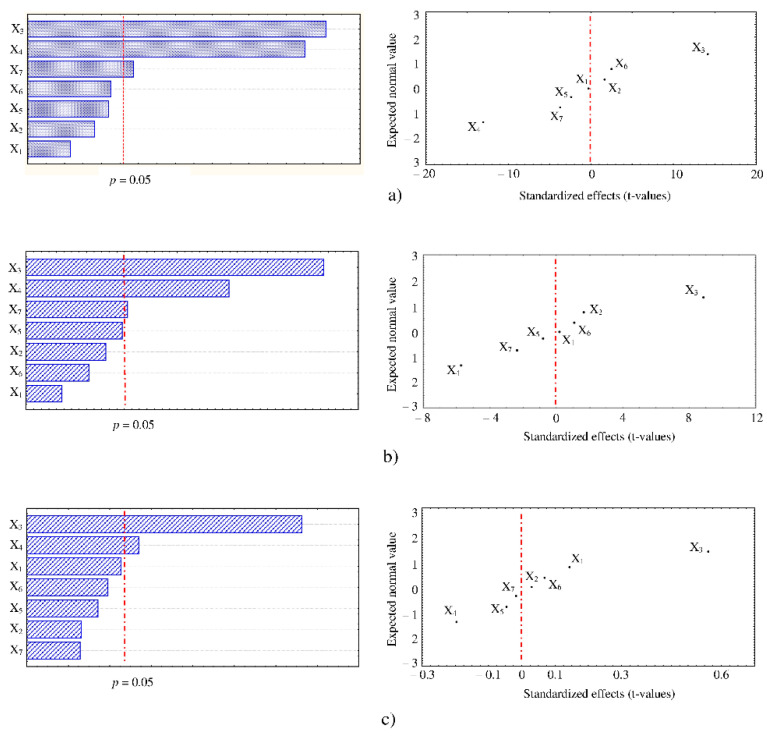
Pareto charts and normal probability plot of standardized effects of seven-factors Plackett–Burman screening design on the extraction of (**a**) gallic acid, (**b**) 4-coumaric acid, and (**c**) myricitrin.

Pareto charts of standardized effects were reported for efficiently illustrating which factor had significative impact on the UAE of the three types of HPLC-DAD identified polyphenols, absorbing at the selected maximum wavelengths; specifically, gallic acid (absorbing at 280 nm), 4-coumaric acid (absorbing at 320 nm), and myricitrin (absorbing at 360 nm). They revealed that solid–solvent ratio (X_3_) and solvent concentration (X_4_) were the most influential factors, while particle size (X_7_) appeared to slightly affect only the extraction recovery of gallic acid and 4-coumaric acid (Figure 2a,b). Moreover, normal probability plots, reporting expected normal values of the variables (i.e., polyphenols) against standardized effects of the factors, showed that X_3_ and X_4_ had positive and negative effect on the polyphenols extraction, as they were distributed on the right and left side, respectively, of the dotted red line (Figure 2a–c). It means that an increase or decrease of polyphenols concentration was observed when X_3_ and X_4_ were changed from lower to higher level. This behavior was similarly reported in a recent study dealing with ultrasound extractions from carob pods, in which solvent concentration and solid to solvent ratio were among the three most dominant factors that influenced the polyphenols recovery, while, conversely to our finding, the other determinant factor was the sonication power [17]. Probably, this difference could be ascribed to the use by Christou et al. [17] of an ultrasonic probe system in their experiment; indeed, a previous research have demonstrated that, when an ultrasonic water bath is employed for extracting D-pinitol from carob pods, the sonication power is not statistically significant [19]. 

As regards the other factors tested in our study, negligible importance of extraction temperature and time was particularly unexpected. Indeed, generally, temperature and time can condition the extraction efficiency by varying the release and diffusion of polyphenols, solvent viscosity and matrix penetration, as well as the starting of oxidation and degradation reactions [17,18,24,25]. The reduced interval of the two factors levels due to the specific screening design applied could just be a partial explanation of this anomaly; however, some controversial interpretation exists in literature on the most suitable values of extraction time and temperature, pending for either lower or higher values of these operating parameters [18,26,27].

In order to reduce the energy costs and provide an extraction of the polyphenol pool as complete as possible according to relevant literature reports, extraction temperature and time and sonication power and frequency were fixed to 35 °C, 15 min, 100 W, and 37 kHz, respectively, in the following optimization process [6,22].

### 3.3. Multi-Response Optimization of UAE by CCD-RSM and D

A non-standard CCD (with α= 1.6818 for rotatability) was chosen for optimizing the above selected three factors (X_3_, X_4_, and X_7_), affecting polyphenols extraction from the ripe carob pods, because it is a better alternative to the full factorial three-level design since it needs a smaller number of experiments while ensuring comparable results [28]. The concentrations of the 9 phenolic compounds (expressed in μg/mL) and the natural values of the factors for the 20 experiments, randomly executed to obtain an accurate estimation of the experimental error, are reported in Table 3, while Table 4 groups the predictive second order polynomial equations, generated applying the quadratic regression models to the Box–Cox transformed experimental values for UAE, in order to describe the empirical relationship between polyphenols concentrations and operational conditions (solid–solvent ratio, solvent concentration, and particle size). 

The reliability of the obtained polynomials was demonstrated by testing the non-significance (*p* > 0.05) of the models lack of fit, performed by repeating six folds the observations at the center point as typically suggested in the case of three-factor CCDs [29]. The determination coefficients (R^2^) were generally >0.8, indicating that just <20% of the total variations was not explained by the models as well as an overall good degree of correlation between the observed and predicted values. Then, the adjusted determination coefficients (R^2^_adj_) were close to R^2^, confirming good statistical models (Table 4). 

**Figure 3:**
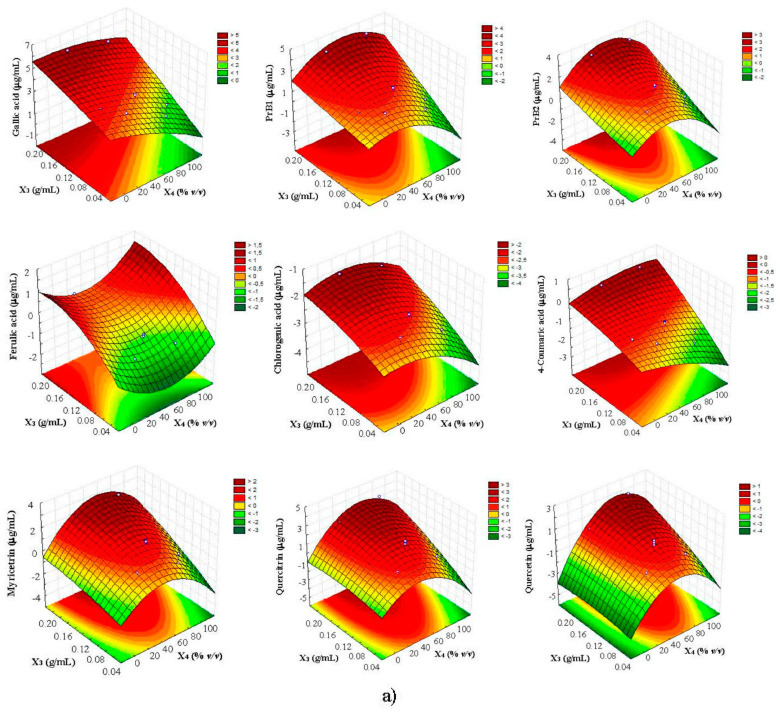

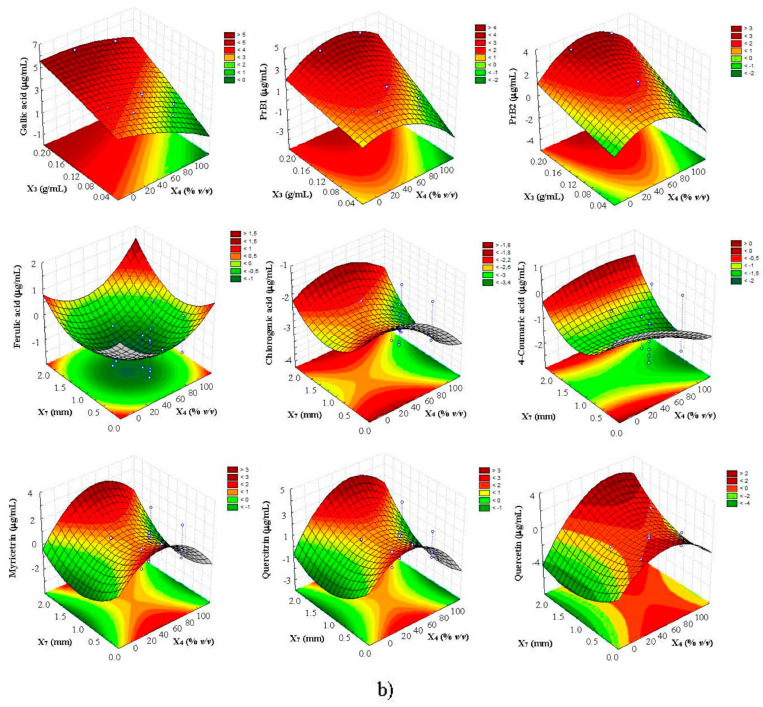
Response surface plots showing the effects of (**a**) solid-solvent (X_3_) vs. solvent concentration (X_4_) and (**b**) particle size (X_7_) vs. solvent concentration (X_4_) on polyphenols recovery from carob pods by UAE.

The linear term of X_3_ was the most significant factor (*p* < 0.01), affecting the extraction of all compounds; when this factor increased from 0.05 to 0.2 g/mL due to the volume change, an increase of polyphenols yield was generally observed, as illustrated by the response surfaces which were generated on the basis of the acquired polynomial equations (Figure 3). Although this finding could be particularly appreciable in term of solvent saving and sustainability, it is worth noting that it appeared in contrast to literature statements; indeed, a decrease of solid–solvent ratio generally results in better swelling of plant material, thus enhancing the mass transfer of polyphenols and, consequently, the yield of extraction [14,17]. However, other authors, dealing with MAE experiments on tea powder, have showed that lower polyphenols recoveries were obtained at lower solid–solvent ratio when the solid mass was maintained constant and the solvent volume changed [30]. 

With regards to X_4_ factor, its quadratic term was significant in the case of procyanidins and, in particular, flavonols (myricitrin, quercitrin, and quercetin), whose values initially increased upon the raise of ethanol percentage and reached a maximum level, after which they started to decrease (Figure 3). This behavior was totally expected because the extraction of phenolic compounds from plant matrix is generally performed with organic solvents (mainly methanol or ethanol) by adding water to create a more polar medium and act as a swelling agent that enables better mass transfer of the bioactive compounds [31]. In particular, the use of middle ethanol concentrations (30–50%) in water enhances the extraction efficiency thanks to the increased solubility of phenolics, especially when non-conventional extraction methods are employed [26,32].

Finally, the quadratic term of X_7_ was also significant, with positive coefficients responsible for the general saddle-shaped response surfaces generated (Figure 3). Therefore, two maximum at higher (2 mm) and lower (0.3 mm) particle sizes were obtained, with prominent recoveries registered in the latter case, that was in agreement with recent researches in which 250 μm fine powder of carob pod were used to optimize polyphenols extraction [17,22].

**Figure 4:**
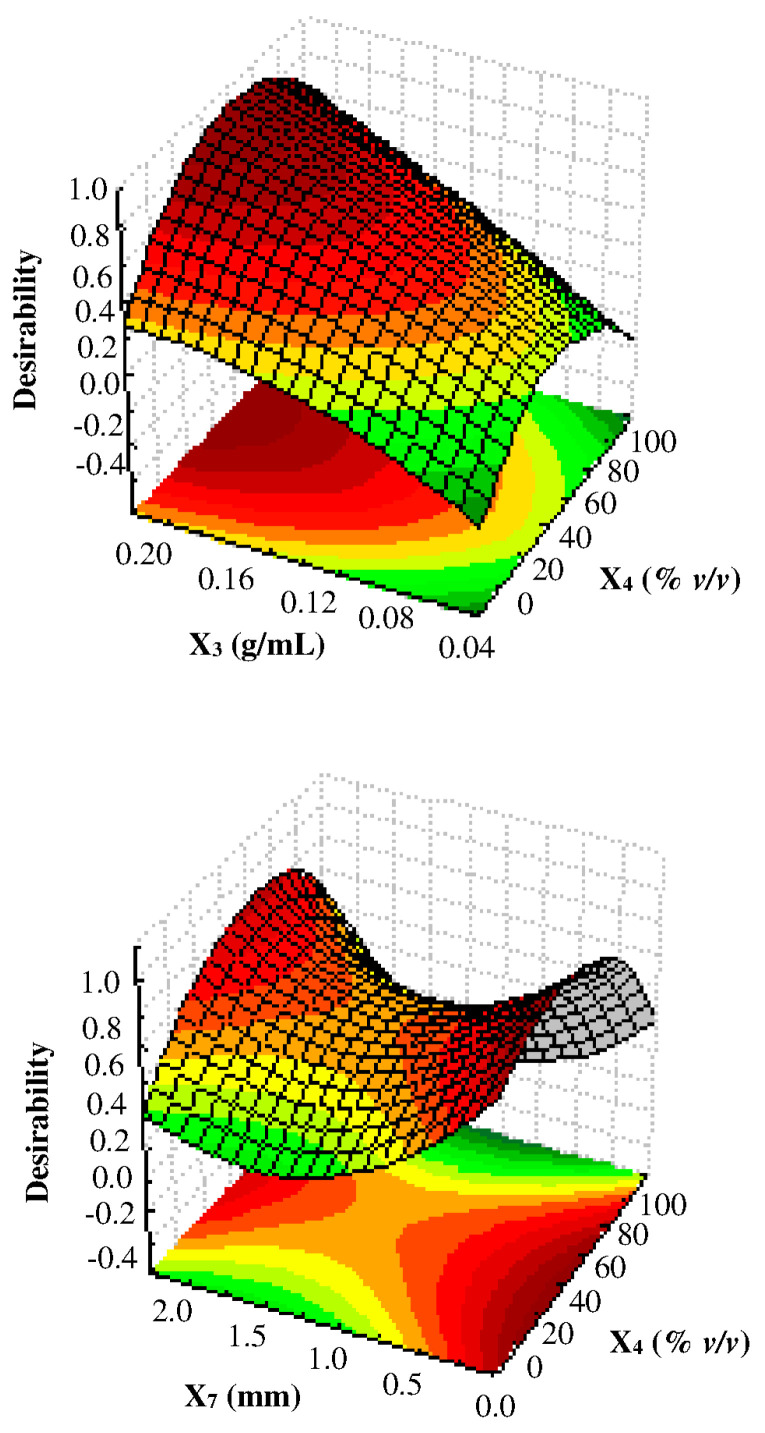
Desirability function (D) for the simultaneous optimization of the 9 polyphenols extracted by UAE from ripe carob. X_3_ = solid–solvent ratio; X_4_ = solvent concentration; X_7_ = particle size.

At this point, because the similarity of the response surfaces (with the exception of ferulic acid), a desirability function [33] was constructed to find the levels of solid–solvent ratio (X_3_), solvent concentration (X_4_), and particle size (X_7_) of the powder carob pod which simultaneously optimize the concentration of the 9 polyphenols extracted by UAE (Figure 4). Briefly, each return of Box–Cox transformed dependent variables was modified over the experimental region into an individual desirability function which ranges between 0 and 100% according to the closeness of the response to undesirable or very desirable values, respectively. In particular, values 15% lower than the maximum or 15% higher than the minimum of each variable have been considered acceptable (desirability 100%) or unacceptable (desirability 0%), respectively. The best experimental conditions derived from the multi-response optimization were as follow: X_3_ = 0.2 g/mL, X_4_ = 40% (*v*/*v*), and X_7_ = 0.3 mm, from which a series of predicted values was obtained (Table 5).

Finally, to check the reliability of the multi-response model, further extraction trials were carried out at the optimal conditions appreciated by the RSMs and D and the gathered experimental data were confronted with the predicted phenolics yield, showing a difference between values lower than 10% (Table 5), which is really in line with other literature studies [14]. The good agreement between the experimental and expected results corroborates the effectiveness and validity of the RSM and D models to ponder the response values and, consequently, to delineate the best extraction conditions.

## 4. Conclusions

This research aimed at obtaining a multi-response optimization of extraction conditions of 9 phenolic compounds, namely 4 phenolic acids (gallic acid, chlorogenic acid, 4-coumaric acid, and ferulic acid), 2 condensed tannins (procyanidins B1 and B2), and 3 flavonols (myricitrin, quercitrin, and quercetin), from carob pods of cv. Amele through non-conventional extraction technology (UAE) using a non-standard CCD coupled to RSM and D statistical tools. The collected results highlighted that the mathematical models built in this study were reliable for the prediction of phenolic compounds extracted from carob pods and that solid–solvent ratio, solvent concentration, and particle size were the three factors conditioning their recovery with the best results obtained at values 0.2 g/mL, 40% ethanol, and 0.3 mm, respectively. Conversely, extraction temperature and time as well as sonication power and frequency did not significantly affect UAE of polyphenols, as highlighted by P-B screening design.

In conclusion, the findings from this study confirm the potential of carob pods as a natural source of polyphenols and contribute to give new insight about their optimal extraction conditions. Furthermore, they propose UAE as an effective and sustainable technology for the revalorization of this agri-food waste.

## Figures and Tables

**Table 1:** Nominal Values of the Independent Variables Used in the Two-Level Plackett–Burman Screening Design.

Factor	Symbol	Factor Levels	
		Low (−1)	High (+1)
Extraction time (min)	X_1_	5	60
Extraction temperature (°C)	X_2_	15	50
Solid–solvent ratio (g/mL)	X_3_	0.05	0.2
Solvent concentration (% ethanol, *v/v*)	X_4_	0	100
Sonication frequency (kHz)	X_5_	37	80
Sonication power (W)	X_6_	30	100
Particle size (mm)	X_7_	0.3	2

**Table 2:** Seven Factors and Two Levels Plackett–Burman (P.-B.) Screening Design.

Run	X_1_ (min)	X_2_ (°C)	X_3_ (g/mL)	X_4_ (% Ethanol *v*/*v*)	X_5_ (kHz)	X_6_ (W)	X_7_ (mm)	Gallic Acid (μg/mL)	4-Coumaric Acid (μg/mL)	Myricitrin (μg/mL)
12(C)	32.5	32.5	0.125	50	60	60	1.0	32.40	0.66	8.59
3	5.0	50.0	0.2	100	80	30	2.0	9.88	0.37	1.60
1	5.0	15.0	0.2	0	80	100	0.3	77.90	1.44	1.94
5	5.0	15.0	0.05	0	37	30	2.0	9.27	0.21	0.44
6	60.0	15.0	0.05	100	80	30	0.3	0.65	0.036	0.17
9(C)	32.5	32.5	0.125	50	60	60	1.0	30.40	0.90	7.66
11(C)	32.5	32.5	0.125	50	60	60	1.0	22.20	0.78	4.52
7	5.0	50.0	0.05	100	37	100	0.3	3.67	0.17	0.67
10(C)	32.5	32.5	0.125	50	60	60	1.0	26.00	0.48	6.69
4	60.0	50.0	0.2	0	37	30	0.3	86.50	2.29	2.94
2	60.0	15.0	0.2	100	37	100	2.0	15.20	0.56	2.86
8	60.0	50.0	0.05	0	80	100	2.0	11.60	0.24	0.59

**Table 3:** Three Level Central Composite Design (CCD) Used for UAE Optimization.

Run	X_3_ (g/mL)	X_4_ (% Ethanol *v*/*v*)	X_7_ (mm)	Gallic Acid (μg/mL)	PrB_1_ (μg/mL)	PrB_2_ (μg/mL)	Ferulic Acid (μg/mL)	ChloroGenic Acid (μg/mL)	4-Coumaric Acid (μg/mL)	Myricitrin (μg/mL)	Quercitrin (μg/mL)	Quercetin (μg/mL)
20(C)	0.08	50	0.5	7.1	2.2	1.8	0.2	0.02	0.2	1.4	2.2	0.7
3	0.20	20	0.3	70.5	15.2	12.7	1.8	0.21	1.5	7.7	12.2	1.6
19(C)	0.08	50	0.5	6.1	2.0	1.7	0.2	0.02	0.17	1.2	2.3	0.5
11	0.04	50	0.5	4.5	1.6	1.5	0.17	0.02	0.12	1.1	1.9	0.4
5	0.05	80	0.3	11.6	3.5	3.2	0.4	0.03	0.4	2.4	2.9	1.0
17(C)	0.08	50	0.5	11.3	3.6	2.9	0.4	0.04	0.30	2.6	4.6	0.6
15(C)	0.08	50	0.5	21.7	7.5	6.7	0.7	0.08	0.6	5.8	13.3	2.8
18(C)	0.08	50	0.5	22.7	7.3	6.5	0.7	0.08	0.6	6.3	11.9	2.5
13	0.08	50	0.3	21.7	7.1	6.9	0.7	0.08	0.6	6.0	10.5	2.1
1	0.05	20	0.3	19.9	4.5	3.7	0.5	0.06	0.5	2.2	3.5	0.8
8	0.2	80	1.0	40.2	16.3	10.0	1.7	0.13	1.3	10.3	9.3	5.2
7	0.2	80	0.3	50.2	13.6	12.9	1.9	0.14	1.7	10.8	13.7	3.4
2	0.05	20	1.0	9.1	2.2	1.7	0.2	0.03	0.2	0.91	1.6	0.13
6	0.05	80	1.0	5.5	1.7	1.6	0.2	0.013	0.19	1.1	1.2	0.4
14	0.08	50	2.0	21.5	7.4	7.0	0.7	0.08	0.6	6.0	13.4	2.5
10	0.08	100	0.5	2.4	0.4	0.5	0.8	0.011	0.10	0.4	0.5	0.3
9	0.08	0	0.5	23.1	4.2	2.0	0.6	0.05	0.5	1.6	1.5	0.01
12	0.21	50	0.5	34.9	10.2	9.4	1.2	0.13	1.0	7.3	10.7	2.3
16(C)	0.08	50	0.5	12.8	3.6	3.2	0.4	0.04	0.4	2.0	1.7	0.7
4	0.2	20	1.0	45.5	10.0	7.4	1.2	0.13	0.9	4.8	7.1	0.7

PrB_1_: Procyanidin B_1_; PrB_2_: Procyanidin B_2_.

**Table 4:** Quadratic Equations for the 9 Compounds Box–Cox Transformed Values Extracted by UAE from Ripe Carob.

Compound	Equation	R^2^	R^2^_adj_	Lack of Fit (*P*)
Gallic acid	4.35 + 11.68X_3_ + 0.38X_7_^2^	0.8418	0.7993	0.6462
Procyanidin B1	2.50 + 7.13X_3_ − 0.04X_4_^2^ + 0.38X_7_^2^	0.8249	0.7674	0.6102
Procyanidin B2	1.35 + 7.38X_3_ − 0.05X_4_^2^ + 0.38X_7_^2^	0.8259	0.7693	0.8053
Ferulic acid	−1.04 + 22.89X_3_ + 0.21X_7_^2^	0.8624	0.8386	0.9947
Chlorogenic acid	−2.38 + 8.28X_3_ − 0.01X_4_^2^ + 0.15X_7_^2^	0.8733	0.8592	0.9719
4-Coumaric acid	−0.45 + 8.22X_3_ + 0.25X_7_^2^	0.8075	0.7343	0.6034
Myricitrin	0.38 + 11.5X_3_ − 0.05X_4_^2^ + 0.34X_7_^2^	0.8058	0.7310	0.8848
Quercitrin	1.18 + 9.98X_3_ − 0.06X_4_^2^ + 0.36X_7_^2^	0.7697	0.6624	0.9149
Quercetin	−1.03 + 6.81X_3_ + 0.56X_4_ − 0.06X_4_^2^ + 0.33X_7_^2^	0.8271	0.7714	0.9617

R^2^ represents the fraction of variation of the response explained by the model; R^2^_adj_ represents the fraction of variation of the response predicted by the model; all *P*-values for the lack of it test obtained in the ANOVA for the quadratic model were not significant (*p* > 0.05); only significant regression coefficients were reported in the equations. X_3_ = solid–solvent ratio; X_4_ = solvent concentration; X_7_ = particle size.

**Table 5:** Content of the 9 Phenolic Compounds in Ripe Carob Extract Obtained at UAE Optimized Conditions (X_3_ = 0.2 g/mL; X_4_ = 40% ethanol/water *v*/*v*; X_7_ = 0.3 mm).

Compound	Experimental (μg/mL)	Predicted (μg/mL)
Gallic acid	56.6 ± 1.5	61.5
Procyanidin B1	14.1 ± 1.2	15.3
Procyanidin B2	13.8 ± 0.8	14.7
Ferulic acid	1.39 ± 0.11	1.51
Chlorogenic acid	0.21 ± 0.04	0.20
4-Coumaric acid	1.47 ± 0.13	1.61
Myricitrin	10.1 ± 1.6	11.0
Quercitrin	14.8 ± 1.9	16.3
Quercetin	2.61 ± 0.17	2.8

Experimental values are expressed as mean ± standard deviation of three replicates; predicted values are generated from the previously optimized models.

## Supplementary Materials

The following supporting information can be downloaded at: https://www.mdpi.com/article/10.3390/foods11030284/s1, Figure S1: Histogram and normality plots of original and Box-Cox transformed data based on the equation y(λ) = (y^λ^ − 1)/λ. (**a**) gallic acid (λ = 0.1552); (**b**) PrB1 (λ = 0.3009); (**c**) PrB2 (λ = 0.2362); (**d**) ferulic acid (λ = 0.0029); (**e**) chlorogenic acid (λ = 0.1512); (**f**) 4-coumaric acid (λ = 0.040); (**g**) myricitrin (λ = 0.1391); (**h**) quercitrin (λ = 0.0672); (**i**) quercetin (λ = 0.3261).
