# Supplementary material for: Ultrasound Assisted Extraction of Polyphenols from Ripe Carob Pods (Ceratonia siliqua L.): Combined Designs for Screening and Optimizing the Processing Parameters
Source: Foods. 2022 Jan 21;11(3):284. doi: 10.3390/foods11030284 (PMC8833885; doi:10.3390/foods11030284)
Supplement: Supplementary file 1 [file foods-11-00284-s001.zip › foods-1522377-supplementary.pdf]

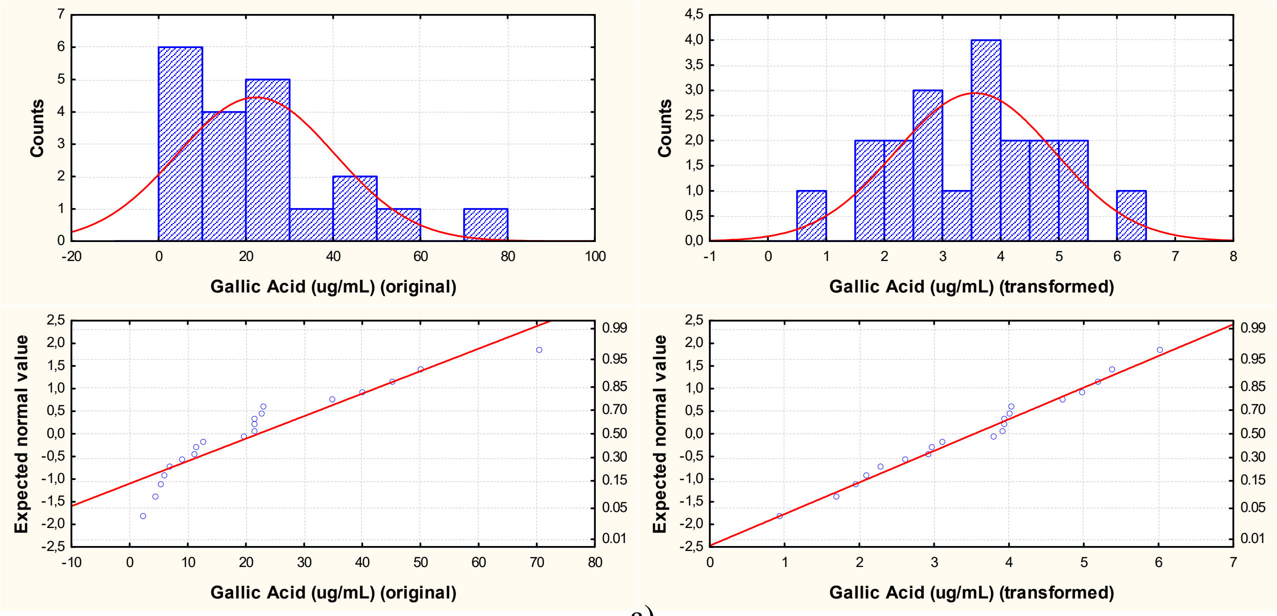
Counts
Gallic Acid (ug/mL) (original)
Counts
Gallic Acid (ug/mL) (transformed)
Expected normal value
Gallic Acid (ug/mL) (original)
Expected normal value
Gallic Acid (ug/mL) (transformed)

a)

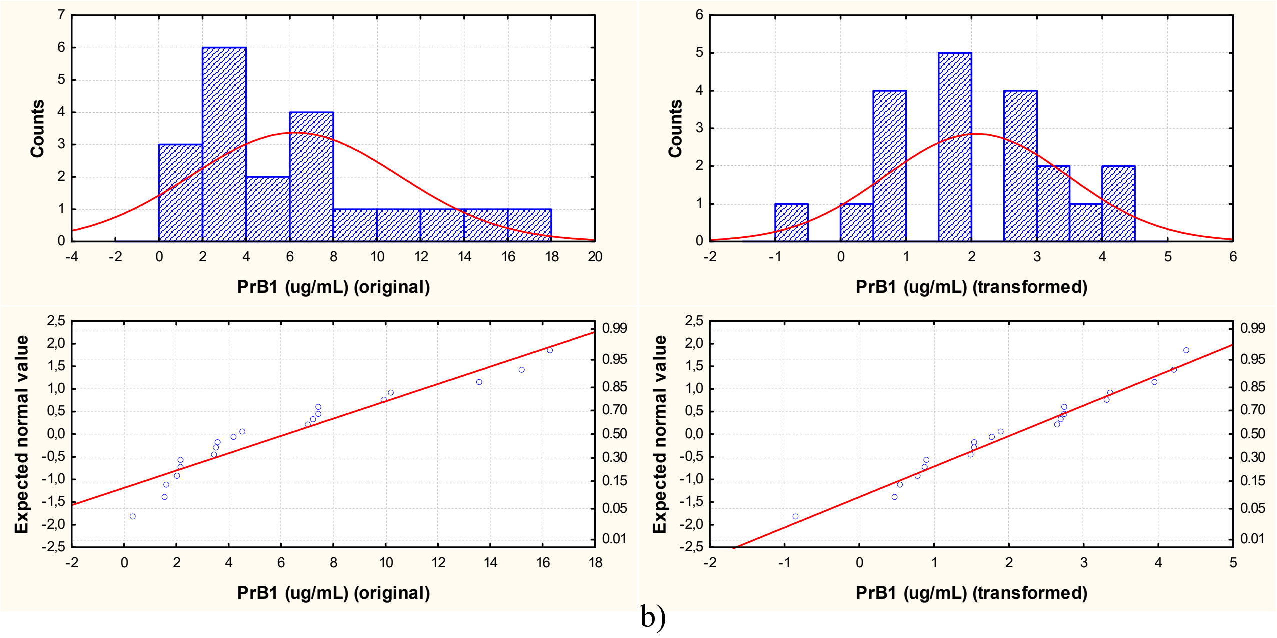
Counts
PrB1 (ug/mL) (original)
Counts
PrB1 (ug/mL) (transformed)
Expected normal value
PrB1 (ug/mL) (original)
Expected normal value
PrB1 (ug/mL) (transformed)

b)

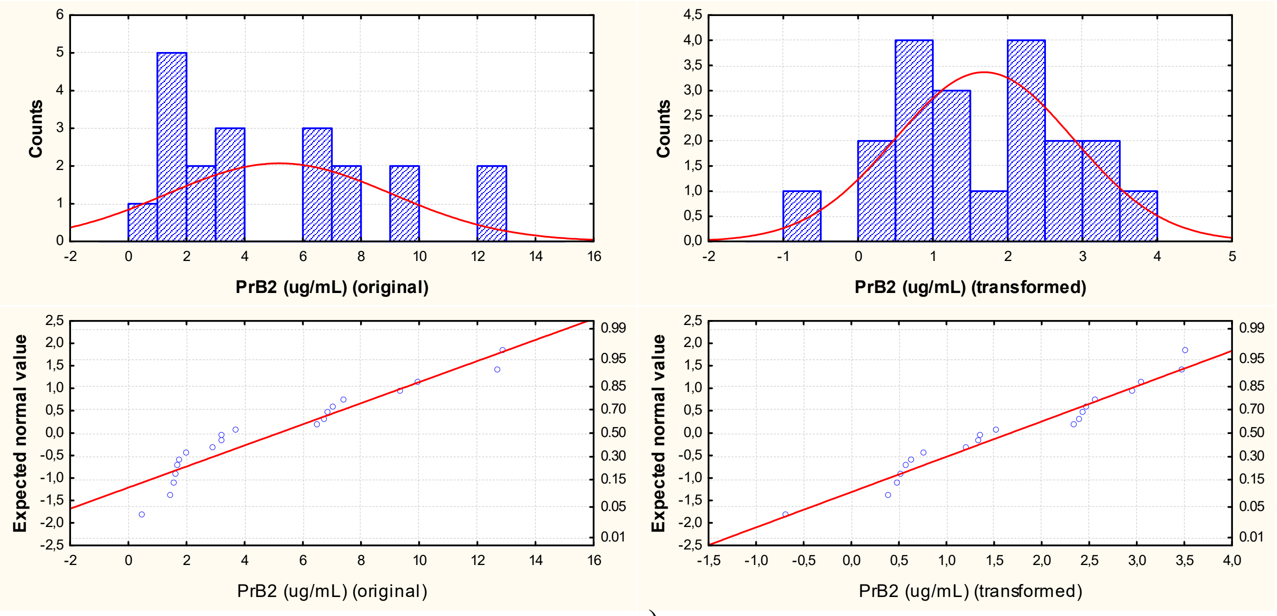
Counts
PrB2 (ug/mL) (original)
Counts
PrB2 (ug/mL) (transformed)
Expected normal value
PrB2 (ug/mL) (original)
Expected normal value
PrB2 (ug/mL) (transformed)

c)

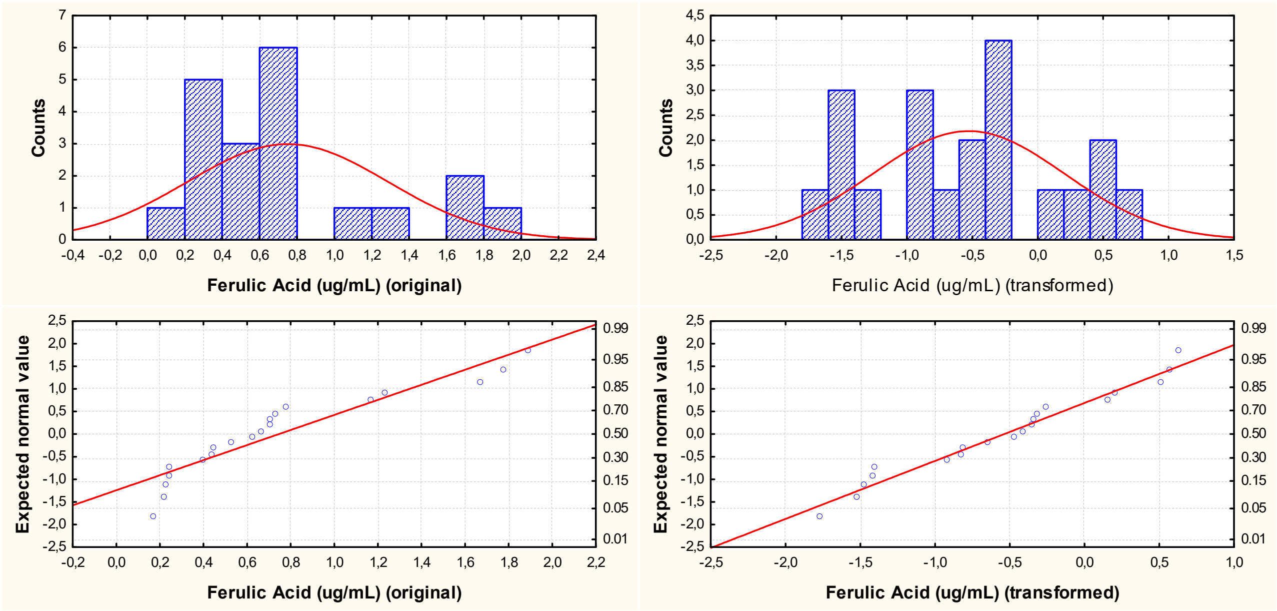
Counts
Ferulic Acid (ug/mL) (original)
Counts
Ferulic Acid (ug/mL) (transformed)
Expected normal value
Ferulic Acid (ug/mL) (original)
Expected normal value
Ferulic Acid (ug/mL) (transformed)

d)

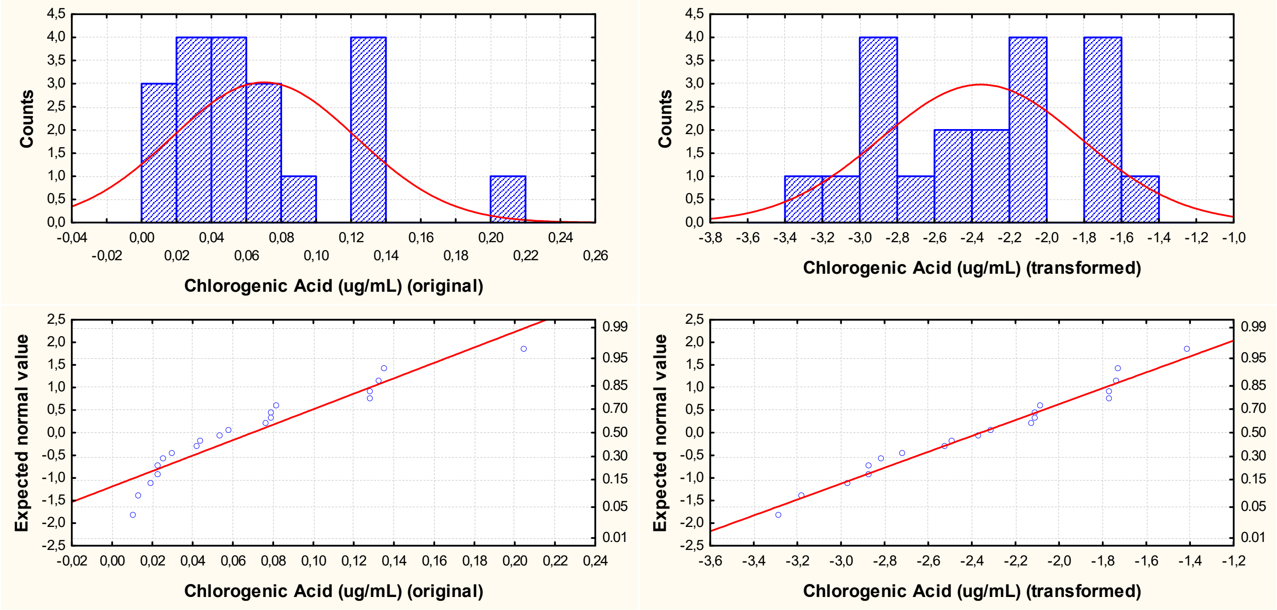
Counts
Chlorogenic Acid (ug/mL) (original)
Counts
Chlorogenic Acid (ug/mL) (transformed)
Expected normal value
Chlorogenic Acid (ug/mL) (original)
Expected normal value
Chlorogenic Acid (ug/mL) (transformed)

e)

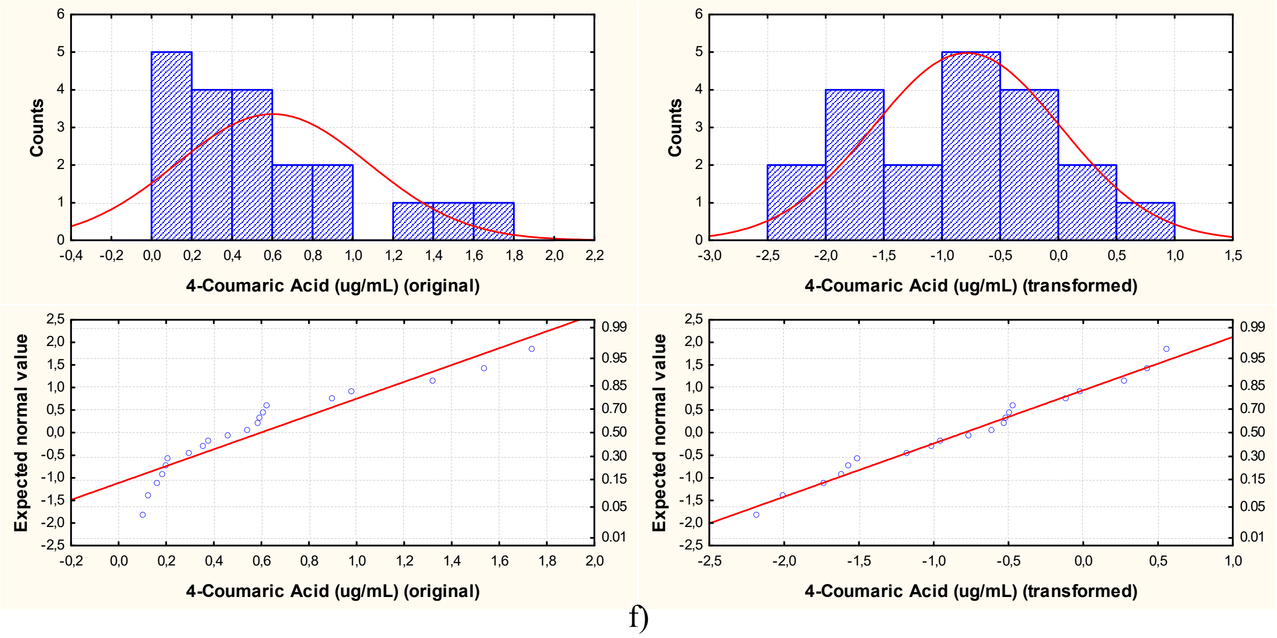
Counts
4-Coumaric Acid (ug/mL) (original)
Counts
4-Coumaric Acid (ug/mL) (transformed)
Expected normal value
4-Coumaric Acid (ug/mL) (original)
Expected normal value
4-Coumaric Acid (ug/mL) (transformed)

f)

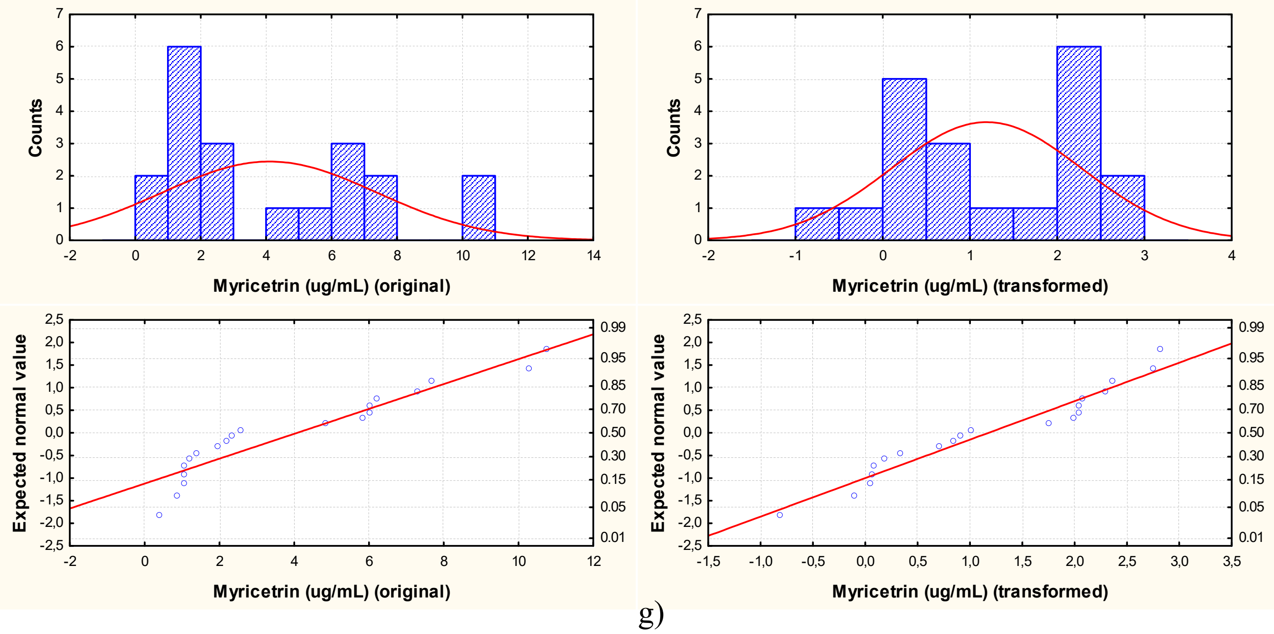

Counts
Myricetrin (ug/mL) (original)
Counts
Myricetrin (ug/mL) (transformed)
Expected normal value
Myricetrin (ug/mL) (original)
Expected normal value
Myricetrin (ug/mL) (transformed)

g)

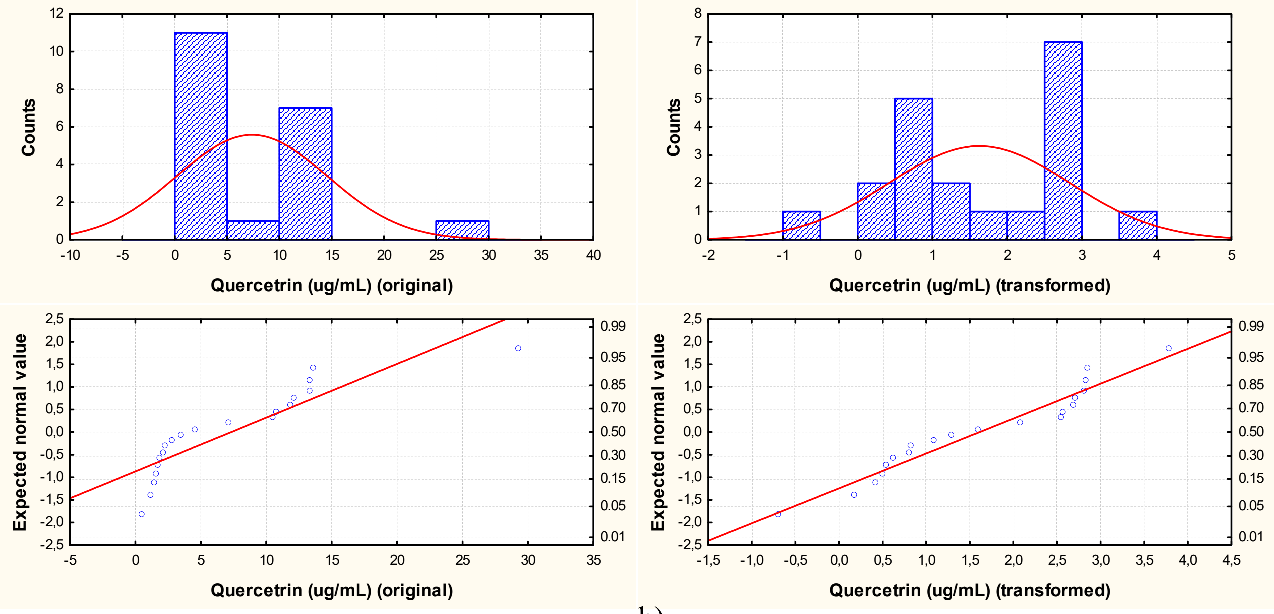

Counts
Quercetrin (ug/mL) (original)
Counts
Quercetrin (ug/mL) (transformed)
Expected normal value
Quercetrin (ug/mL) (original)
Expected normal value
Quercetrin (ug/mL) (transformed)

h)

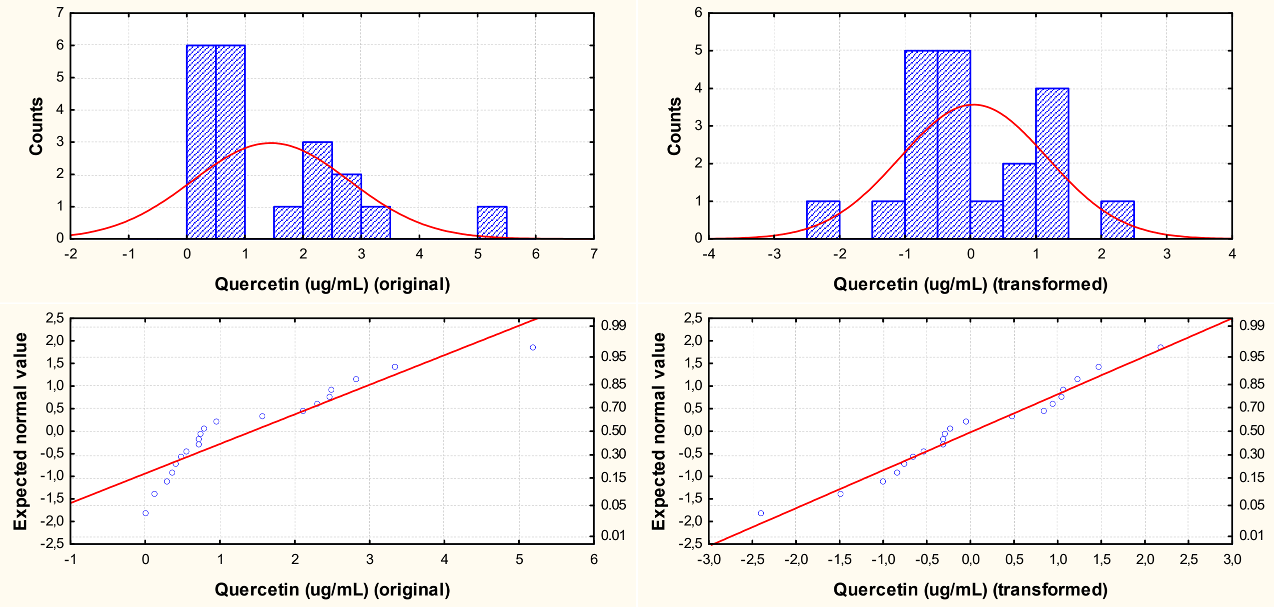

Counts
Quercetin (ug/mL) (original)
Counts
Quercetin (ug/mL) (transformed)
Expected normal value
Quercetin (ug/mL) (original)
Expected normal value
Quercetin (ug/mL) (transformed)

i)

**Figure S1.** Histogram and normality plots of original and Box-Cox transformed data based on the equation $y(\lambda) = (y^{\lambda} - 1)/\lambda$. a) gallic acid ($\lambda = 0.1552$); b) PrB1 ($\lambda = 0.3009$); c) PrB2 ($\lambda = 0.2362$); d) ferulic acid ($\lambda = 0.0029$); e) chlorogenic acid ($\lambda = 0.1512$); e) 4-coumaric acid ($\lambda = 0.040$); g) myricitrin ($\lambda = 0.1391$); h) quercitrin ($\lambda = 0.0672$); i) quercetin ($\lambda = 0.3261$).
